# The efficacy of extracorporeal membrane oxygenation in liver transplantation from non-heart-beating donors: A systemic review and meta-analysis

**DOI:** 10.1097/MD.0000000000014722

**Published:** 2019-03-01

**Authors:** Jiang-Chen Peng, Jia Ding, Zheng-Yu He, Yu-Xiao Deng, Shun-Peng Xing, Xian-Yuan Zhao, Zhe Li, Yi-Li Dai, Yuan Gao

**Affiliations:** Department of Critical Care, Ren Ji Hospital, School of Medicine, Shanghai Jiao Tong University, Shanghai, China.

**Keywords:** 1-year graft survival rate, 1-year patient survival rate, donors after brain death, extracorporeal membrane oxygenation, non-heart-beating donors

## Abstract

**Background:**

A systematic review and meta-analysis was made to see whether extracorporeal membrane oxygenation (ECMO) in liver transplantation could improve non-heart-beating donors (NHBDs) recipients’ outcomes compared with donors after brain death (DBDs) recipients.

**Methods:**

We searched MEDLINE, EMBASE, and Cochrane Central Register of Controlled Trials for eligible studies. The study eligible criteria are cohort or case–control studies using ECMO in all NHBDs; studies involved a comparison group of DBDs; and studies evaluated 1-year graft and patient survival rate in NHBDs and DBDs groups.

**Results:**

Four studies with 704 patients fulfilled the inclusion criteria. The pooled odds ratio (OR) of 1-year patient survival rate in NHBDs recipients compared with DBDs recipients was 0.8 (95% confidence interval [CI], 0.41–1.55). The pooled OR of 1-year graft survival rate in NHBDs recipients compared with DBDs recipients was 0.46 (95% CI, 0.26–0.81). NHBDs recipients were at greater risks to the occurrence of primary nonfunction (PNF) (OR = 7.12, 95% CI, 1.84–27.52) and ischemic cholangiopathy (IC) (OR = 9.46, 95% CI, 2.76–32.4) than DBDs recipients.

**Conclusions:**

ECMO makes 1-year patient survival acceptable in NHBDs recipients. One-year graft survival rate was lower in NHBDs recipients than in DBDs recipients. Compared with DBDs recipients, the risks to develop PNF and IC were increased among NHBDs recipients.

## 1 Introduction

Organ transplantation is the most cost-effective treatment for end-stage organ failure, which improves the quality of life and increases the life expectancy of patients. However, even in countries with high donation rates such as Spain, the donor pool is not able to meet the demand for transplantable organs. Based on the prevalence of organ transplantation and shortage of donor organs, it is necessary to expand the criteria for existing pools. So the focus of transplantation center has turned to donation after circulatory death (DCD).

DCD donors were classified into 4 categories according to Maastricht criteria^[1]^: dead upon arrival at the hospital and not resuscitated, unsuccessful resuscitation attempt, cardiac arrest (CA) after removing ventilatory support from a patient with brain damage insufficient to declare brain death, and unanticipated CA following the diagnosis of brain death. Types 1, 2, and 4 are considered as uncontrolled non-heart-beating donors (UNHBDs) and type 3 is called controlled non-heart-beating donors (CNHBDs). Recently, DCD has garnered significant interest and the number of DCD donors has increased progressively over the past decade. However, the weakness of DCD organs is warm ischemia during hypotensive phase after removing ventilatory support and cardiac arrest.^[2]^ Besides, ischemia/reperfusion injury could also cause severe damage to the DCD organs. Several pathways, such as inflammatory response, oxygen free radicals, and activation of T-cell lymphocytes, have been involved in ischemia/reperfusion injury.^[3]^ These are the main factors that result in higher incidence of primary nonfunction (PNF) and delayed graft function (DGF) in kidney transplantation,^[4]^ graft loss, and biliary complications in liver transplantation.^[5]^ So, traditional preservation method based on hypothermic storage is not suitable for DCD organs. Because cold storage only aggravates the grafts which have already suffered from hypoxia and hypoperfusion,^[6]^ new preservation techniques are needed to stop or even reverse the cellular injury.

Regional perfusion by extracorporeal membrane oxygenation (ECMO) seems to be a promising method for perfusing organs from DCD. It is used to recirculate the donor's own oxygenated blood to support single organ rather than to use another preservation solution. It works as a perfusion bridge during asystole and procurement period which decreases the risk of ischemic damage. Besides, by maintaining organs under physiological conditions, it replenishes mitochondrial stores of adenosine triphosphate (ATP) which enables rehabilitation on a cellular level.^[7,8]^

To date, there has been no systemic review based on regional perfusion by ECMO in the application among NHBDs in liver transplantation. The aim of this study is to systemically review the role of ECMO in NHBDs liver transplantation and make meta-analysis to compare its efficacy with DBDs.

## 2 Materials and methods

### 2.1 Search strategy

This review was performed according to the standard guidelines for meta-analyses and systematic reviews of observational studies.^[9]^ To find relevant articles for this review, we searched the following databases (from inception to December 2017): MEDLINE, EMBASE, and Cochrane Central Register of Controlled Trials. The search strategy used free-text words and Medical Subject Headings terms to increase the sensitivity of the search. The following search terms were used: *liver transplantation*, *extracorporeal membrane oxygenation*, *regional perfusion*, *non-heart-beating donors*, *donors after circulatory death*, and *donors after cardiac death*. Boolean operators (AND, OR, NOT) were used to ensure a comprehensive review.

### 2.2 Inclusion and exclusion criteria

For inclusion in the systematic review, a study had to meet the following criteria established by the study team: cohort or case–control studies using ECMO in all NHBDs; studies involved a comparison group of DBDs; and studies evaluated 1-year graft and patient survival rate in NHBDs and DBDs groups.

Studies were excluded if studies were case reports, review articles, animal studies, and studies of other organs; studies lacked a control group of DBDs; and articles not written in English were excluded.

## 3 Data extraction

To reduce reporting bias and error in data collection, all papers were examined independently for eligibility by 2 reviewers (Peng and Ding). Disagreement was resolved by consulting a third reviewer (Gao). Standardized data extraction form created by the study team was used. This form included the authors, location, year of publication, study design, donor ages, Maastricht categories, circuit temperature, donor warm ischemia time (DWIT), cold ischemia time, RP duration, number of NHBDs recipients, and number of DBDs recipients.

Methodological quality of included studies was evaluated by Newcastle–Ottawa scale (NOS). It uses a “star” rating system to judge quality on the basis of 3 aspects of the study: selection of study groups, comparability of study groups, and assessment of the exposure. This scale awards a maximum of 9 stars to each study: up to 4 for selection of participants, 2 for comparability of participants, and 3 for assessment of exposure. Studies with scores of 0 to 3, 4 to 6, and 7 to 9 were considered to be low-, moderate-, and high-quality studies.

### 3.1 Statistical analysis

The primary outcome of this analysis was the odds ratio (OR) of 1-year patient and graft survival rate in NHBDs recipients versus DBDs recipients. We calculated the OR with a 95% confidence interval (CI) based on a fixed-effects model using the methods of DerSimonian and Laird.^[10]^ Heterogeneity between the studies was assessed by Chi-square test and the *I*^2^-statistic.^[11]^*P* values <.05 were considered statistically significant. If significant heterogeneity exists, it would be inappropriate to combine the data for further analysis using a fixed-effects model, whereas the random model was used for calculations. Any heterogeneity identified would prompt subgroup analysis in an attempt to explain these findings. Statistical analysis was performed with the software REVMAN X6 from the Cochrane Collaboration.

## 4 Results

### 4.1 Literature search

**Figure 1:**
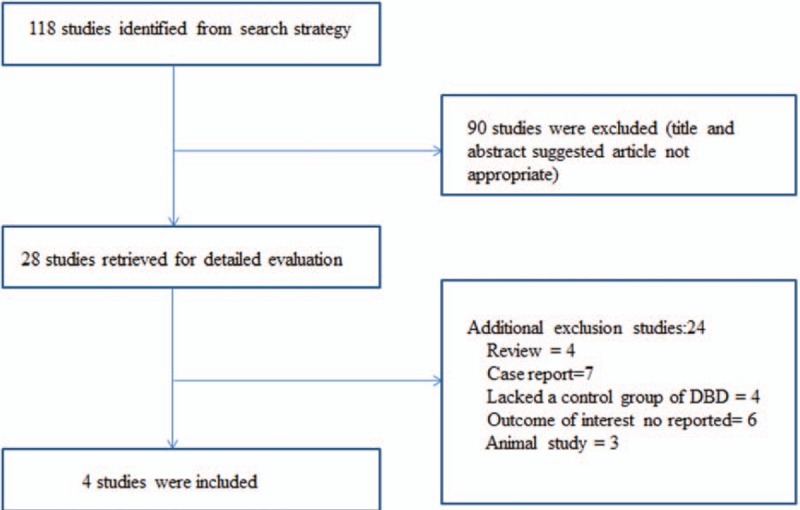
Flow diagram of studies identified in the systemic review.

The search strategy identified 118 citations. After analysis of selected articles, 28 articles were reviewed in detail. Subsequently, 24 articles did not meet the inclusion criteria. The reasons for exclusion included the following: 4 articles were review,^[12–15]^ 7 studies were case reports,^[16–22]^ 4 studies lacked a control group of DBDs ,^[23–26]^ 6 studies did not report the outcome of interest,^[27–32]^ and 3 articles were animal study.^[33–35]^ Therefore, 4 studies^[2,36–38]^ with 704 patients fulfilled the inclusion criteria (Fig. 1).

The characteristics of the included studies are listed in Table 1. All of the studies were conducted in Spain. The NOS of the included studies were all ≥7, which were considered as high quality. Organs were transplanted from Maastricht category II NHBDs in 3 studies,^[36–38]^ whereas one study^[2]^ used organs from Maastricht category III NHBDs. The perfusion temperature in 3 studies was normothermia,^[2,37,38]^ whereas one study evaluated both hypothermia and normothermia perfusion temperature.^[36]^

**Table 1:**
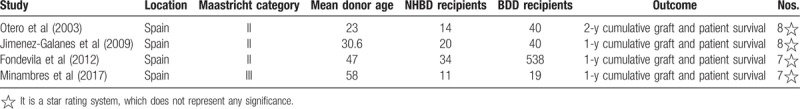
The characteristics of the included studies.

In each study, the femoral artery and vein were cannulated and connected to ECMO. The opposite femoral artery was cannulated with a balloon catheter, which was inflated at the supraceliac aorta to prevent brain and coronary perfusion during RP. The chest radiograph was obtained to ensure the proper positioning of the balloon. For category II NHBDs, cannulae were placed postmortem after a 5-minute standoff period. For category III NHBDs, cannulae were placed under local anesthesia before the withdrawal of mechanical ventilator. The different parameters of RP protocols in each study were displayed in Table 2.

**Table 2:**
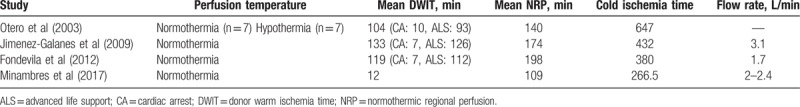
protocol parameters of regional perfusiom.

### 4.2 1-year patient and graft survival rate in NHBDs and DBDs recipients

**Figure 2:**
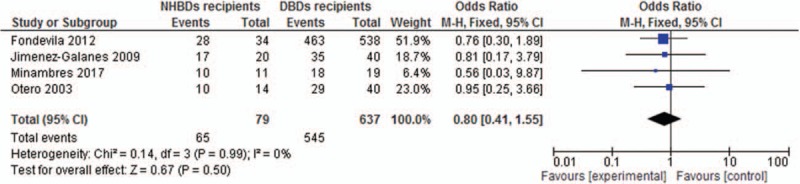
Odds ratio of 1-year patient survival for NHBDs recipients versus BDDS recipients.

Overall, the 1-year patient survival rate in NHBDs and DBDs recipients was 82.3% and 85.6%, respectively. The pooled OR of 1-year patient survival rate in NHBDs recipients compared with DBDs recipients was 0.8 (95% CI, 0.41–1.55) with no statistically significant heterogeneity (*I*^2^ = 0%, *P* = .99) (Fig. 2).

**Figure 3:**
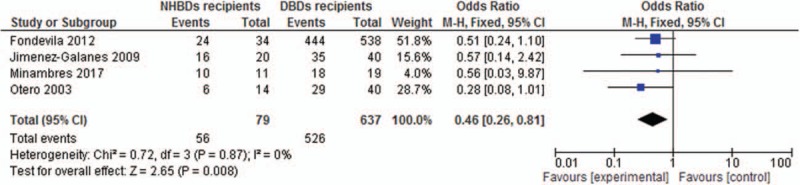
Odds ratio of 1-year graft survival for NHBDs recipients versus BDDS recipients.

The 1-year graft survival rate in NHBDs recipients was lower than that in DBDs recipients (70.9% vs 82.6%). And, the pooled OR of 1-year graft survival rate in NHBDs recipients compared with DBDs recipients was 0.46 (95% CI, 0.26–0.81) with no statistically significant heterogeneity (*I*^2^ = 0%, *P* = .87) (Fig. 3).

### 4.3 Occurrence of complications in NHBDs and DBBs recipients

**Figure 4:**
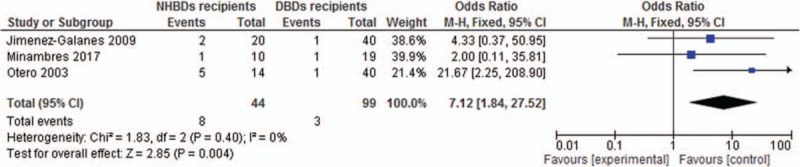
Odds ratio of incidence of PNF for NHBDs recipients versus BDDS recipients.

**Figure 5:**
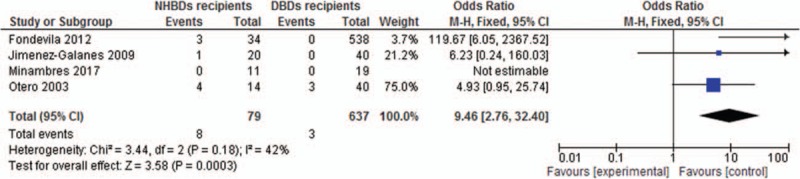
Odds ratio of incidence of IC for NHBDs recipients versus BDDS recipients.

Three of the 4 studies reported the number of patients suffered from PNF. NHBDs recipients were at greater risk to the occurrence of PNF than DBDs recipients (OR = 7.12, 95% CI, 1.84–27.52) (Fig. 4). In terms of ischemic cholangiopathy (IC), NHBDs recipients also had increased risk in the occurrence of IC compared with DBDs recipients (OR = 9.46, 95% CI, 2.76–32.4) (Fig. 5).

## 5 Discussion

During the last decade, the number of liver donors could not meet the growing demand of orthotopic liver transplantation. To reduce the morbidity and mortality in patients with end-stage liver disease, some centers have accepted NHBDs. The early results with NHBDs were unsatisfactory. NHBDs have been related with higher rates of primary nonfucntion, IC, and retransplantation. In 1995, Casadevilla et al^[39]^ from Pittsburgh reported their experience with 6 UNHBDs transplants. Five of them failed because of postoperative complications. They also reported lower graft (17%) and patient (67%) survival. In current years, new methods to expand the donor pool have emerged in use. Regional perfusion by extracorporeal membrane oxygenation seems to be a promising choice. In this study, we systemically review the current literature to evaluate the value of ECMO in NHBDs liver transplantations. We included 4 studies comparing 1-year patient and graft survival between NHBDs and DBDs groups. Three of them focused on UNHBDs transplants, and the other one was concerned about CNHBDs transplants. The results showed that the overall 1-year patient survival between NHBDs and DBDs recipients was similar (0.8, 95% CI, 0.41–1.55). However, the overall 1-year graft survival in NHBDs recipients was significantly lower than that in DBDs recipients (0.46, 95% CI, 0.26–0.81). Although ECMO could be a promising method for perfusing organs from DCDs, there were several other factors that could influence the outcome of the grafts. The most important factor that caused graft failure was the warm ischemia that the graft experienced during resuscitation.^[40]^ Jimenez-Galanes et al^[37]^ concluded that both AST and ALT peaks were significantly higher in UNHBDs group versus DBDs group, which reflected the effect of warm ischemia. Fondevila et al^[38]^ divided the 34 recipients into two-halves. Graft survival improved from the first half to the second. One reason was due to replacement of manual chest compressions with mechanical ones, which increased the perfusion pressure and reduced the damage caused by warm ischemia. Second, the duration of regional perfusion could also result in graft damage. In the study of Otero et al,^[36]^ their results showed that the duration of cardiopulmonary bypass was significantly higher in failing grafts compared with functioning grafts (225 vs 110 minutes, *P* < .01). No graft failed if the duration of cardiopulmonary bypass was <150 min. Besides, Fondevila et al^[38]^ proposed that high-risk recipients may be not appropriate to liver grafts from NHBDs. In their study, the first half of recipients had more Child-Turcotte-Pugh C patients (71% vs 35%, *P* = .039) than the second half. What is more, donor selection was also very important. The lower survival rate may be explained by their less stringent donor criteria and greater acceptance of suboptimal grafts. The British Transplantation Society has deemed liver grafts to be suboptimal when there is >10% hepatic steatosis and the donor age is >50 years.^[41]^ Hepatic steatosis <30% was deemed acceptable by Otero et al.^[36]^ Grafts with unsatisfactory macroscopic appearance related to hepatic steatosis, fibrosis, or congestion were rejected in Fondevila et al's study,^[38]^ but the proportion was not mentioned. However, the more stringent criteria on hepatic steatosis by Jimenez-Galanes et al's study may account for their superior outcomes.^[37]^ Last but not least, the use of thrombolytics may improve the applicability of NHBDs liver transplantation. During cardiac arrest, the formation of microthrombi could result in persistent ischemia even when gross blood flow was restored. If we used thrombolytics during ECMO, we could potentially restore hepatic microvascular circulation and improve graft viability. Investigations are needed to confirm this.^[42]^

With regard to controlled NHBDs liver transplantation, Minambres et al^[2]^ showed that recipients’ outcomes with grafts from controlled NHBDs to ECMO had no significant difference with those obtained from DBDs. The advantage of ECMO was that it could expand the controlled NHBDs’ age with safety. ECMO also allowed the performance of graft retrieval without speed, so it reduced the rate of iatrogenic injuries.

In terms of complications, NHBDs groups had higher risks to develop primary nonfucntion (OR = 7.12, 95% CI, 1.84–27.52) and ischemic chonlangiopathy (OR = 9.46, 95% CI, 2.76–32.4). The arrangement of liver transplantation was not initiated before a liver biopsy from the harvested organ indicated the viability of the graft. So, it took a longer period for organs from NHBDs to prepare for transplantation. Prolonged cold ischemia has been related to biliary strictures in organs from NHBDs^[43]^, which resulted in increased frequency of complications in NHBDs group. What is more, Taner et al^[44]^ from Mayo demonstrated that the time interval from incision to anhepatic phase had a significant impact on graft survival. The placement of balloon confirmed by X-ray in our studies certainly takes additional time, which may decrease the graft survival rate.

There are also some limitations in this study. The significant limitation is the small number of included studies. We still need more studies to further evaluate the role of ECMO in NHBDs liver transplantation. Second, the absence of transplantation experience in early studies also has an impact on the result. What is more, studies with long-term follow-up are needed to see whether ECMO could improve patients’ future surviving state. Besides, the liver transplantations data were all form Spain. We still need more results from other countries to make a comprehensive analysis.

In conclusion, as an emerging technique in liver transplantation, ECMO enables the expansion of donor pool and makes 1-year patient survival acceptable. But we have to consider the increased risks of PNF and IC after NHBDs liver transplantation. However, the application of ECMO in controlled NHBDs liver transplantation is promising. With growing experience and more stringent criteria, NHBDs liver transplantation by ECMO will achieve satisfactory results.

## Author contributions

**Data curation:** Jia Ding, Zheng-Yu He.

**Formal analysis:** Zheng-Yu He.

**Investigation:** Yu-Xiao Deng.

**Methodology:** Jia Ding, Yu-Xiao Deng, Zhe Li.

**Project administration:** Shun-Peng Xing.

**Resources:** Xian-Yuan Zhao.

**Software:** Xian-Yuan Zhao, Yi-Li Dai.

**Writing – original draft:** Jiang-Chen Peng.

**Writing – review and editing:** Yuan Gao.
